# Biology of cyclooxygenase-2: An application in depression therapeutics

**DOI:** 10.3389/fpsyt.2022.1037588

**Published:** 2022-11-10

**Authors:** Ying He, Yuanshan Han, Xiaolin Liao, Manshu Zou, Yuhong Wang

**Affiliations:** ^1^Institute of Innovation and Applied Research in Chinese Medicine, Hunan University of Chinese Medicine, Changsha, China; ^2^Department of Scientific Research, The First Hospital of Hunan University of Chinese Medicine, Changsha, China; ^3^Hunan Provincial Key Laboratory for the Prevention and Treatment of Depressive Diseases with Traditional Chinese Medicine, Changsha, China; ^4^Hunan Key Laboratory of Power and Innovative Drugs State Key Laboratory of Ministry Training Bases, Changsha, China

**Keywords:** COX-2, depression, pro-inflammatory cytokines, mechanism of action, COX-2 inhibitors

## Abstract

Depressive Disorder is a common mood disorder or affective disorder that is dominated by depressed mood. It is characterized by a high incidence and recurrence. The onset of depression is related to genetic, biological and psychosocial factors. However, the pathogenesis is still unclear. In recent years, there has been an increasing amount of research on the inflammatory hypothesis of depression, in which cyclo-oxygen-ase 2 (COX-2), a pro-inflammatory cytokine, is closely associated with depression. A variety of chemical drugs and natural products have been found to exert therapeutic effects by modulating COX-2 levels. This paper summarizes the relationship between COX-2 and depression in terms of neuroinflammation, intestinal flora, neurotransmitters, HPA axis, mitochondrial dysfunction and hippocampal neuronal damage, which can provide a reference for further preventive control, clinical treatment and scientific research on depression.

## Introduction

Depression is a mental disorder. It is often characterized by depressed mood, loss of interest, decreased energy, self-denial, diminished ability to think, and sleep disturbances, etc. According to statistics, 350 million people worldwide suffer from varying degrees of depression, and more than 1 million people commit suicide each year because of depression (1). The pathogenesis of depression is complex, and current pathogenesis includes the monoamine hypothesis, changes in the hypothalamic-pituitary-adrenal axis, the inflammation hypothesis, neuroplasticity and neurogenesis, changes in brain structure and function, and the interaction and influence of genes and the environment (2). Recent studies have revealed the important role of the inflammation hypothesis, especially neuroinflammation, in the pathogenesis of depression (3).

Cyclo-oxygen-ase (COX) is an inflammatory mediator that regulates the inflammatory response and a key rate-limiting enzyme in the initial steps of prostaglandin (PGs) synthesis (4). PGs have a central role in the induction of the inflammatory response and can be synthesized in inflamed and damaged tissues, thus they are associated with the development of acute inflammatory swelling and painful symptoms (5). Among them, PGI2 and PGE2 are involved in vascular permeability, tissue swelling and gastric mucus secretion, which are typical symptoms of inflammation (6). COX has three isoforms, COX-1, COX-2 and COX-3 (6), of which COX-2 is usually induced by inflammatory stimuli in most tissues and is the only isoform responsible for propagating the inflammatory response. Current studies have demonstrated that COX-2 activation is an important factor mediating the development of depression (7, 8). Here, the present study summarizes recent research advances to further describe the link between depression and COX-2, aiming to provide new therapeutic ideas for the treatment of depression.

## Cyclo-oxygen-ase-2 roles in the pathogenesis of depression

### Neuroinflammation

Inflammation is a defense response of the immune system against tissue damage caused by inflammatory factors such as biological, physical, or chemical factors, foreign bodies, or necrotic tissue. One of the inflammatory responses in the brain is called “neuroinflammation.” Neuroinflammation is a protective response of the central nervous system (CNS), which is composed of neurons, microglia, oligodendrocytes and astrocytes. The entry of any foreign pathogen activates glial cells (astrocytes and microglia) and the over-activation of these cells triggers the release of various neuroinflammatory markers (NM), such as tumor necrosis factor-α (TNF-α), interleukin-1β (IL- 1β), interleukin-10 (IL-10), nitric oxide (NO), COX-2, etc. Various studies have shown the importance of neuroinflammatory markers in the development, diagnosis and treatment of depression (9–11).

**FIGURE 1:**
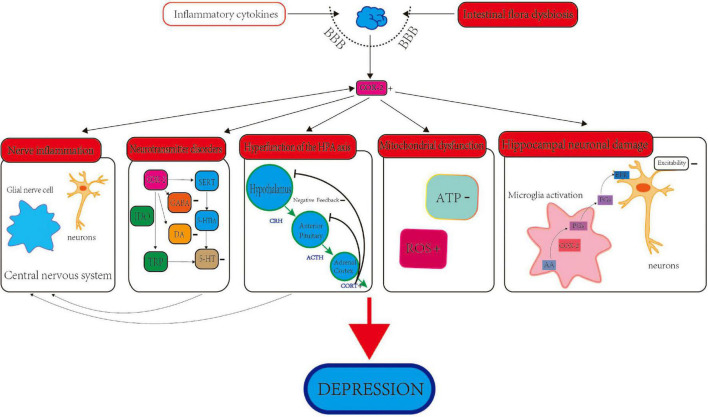
The role and mechanism of COX-2 in depression. The role of COX-2 and depression involves several aspects, including neuroinflammation, intestinal flora, neurotransmitters, HPA axis, deregulation of mitochondrial functions and neuronal damage of hippocampus.

In recent years, an increasing number of scholars have found that neuroinflammation is an important pathological factor triggering central neurological disorders such as depression, and inhibiting central neuroinflammation is a key way to effectively alleviate the onset and progression of depression (12–16). It was found that upregulation of pro-inflammatory cytokines (IL-6 and TNF-α) was seen in the hippocampus and cortex of Lipopolysaccharide (LSP)-induced depressed mice (17). Inflammatory cytokines in the blood can activate the hypothalamic-pituitary-adrenal (HPA) axis, and HPA dysfunction leads to corticotropin releasing hormone (CRH), adrenocorticotropic hormone (ACTH), increased secretion of corticosterone (CORT) and its negative feedback dysfunction, upregulate the release of glucocorticoids(GC) and cortisol in the adrenal cortex as well as cause dysregulation of monoamine transmitters, while inflammatory factors decrease the release of glucocorticoids and cortisol through activation of indoleamine-2,3-dioxygenase (IDO) enzyme, IDO activation reduces tryptophan and increases toxic metabolites of the kynurenine pathway, which leads to damage to astrocytes, microglia, and neurons, triggering neuroinflammation, which is positively associated with Major Depressive Disorder (MDD) (18–20). In addition, inflammatory factors can also induce damage to the blood-brain barrier (BBB) and enter the brain (21, 22). Damage to the hippocampal BBB, an important portal for maintaining a homeostatic environment for neurons and glial cells, would allow increased permeability and trigger neuroinflammation (23), which may be an important pathological factor in triggering depression (24). COX-2 is expressed in the central nervous system and is associated with neuroinflammation (25). Prabhakaran et al. used PET imaging to directly measure brain COX-2 levels, which showed that COX-2 can be induced to be upregulated during neuroinflammation, causing elevated COX-2 levels *in vivo* (26). The anti-neuroinflammatory effect is mediated by modulation of COX-2 levels to produce PGE2. The novel GPR55 receptor antagonist KIT10 may reduce neuroinflammation in microglia by inhibiting COX-2/PGE2. It has been shown that KIT10 inhibits the onset of depression by inhibiting protein synthesis of mPGES-1 and COX-2 and reducing PGE2 levels (26, 27). Neuroinflammation can mediate the onset of depression by affecting the reduction of monoamine neurotransmitters or changes in the number and sensitivity of their receptors. Venlafaxine, a serotonin-norepinephrine reuptake inhibitor, is a clinical agent used in the treatment of depression. It has anti-injurious and anti-inflammatory activity by inhibiting pro-inflammatory cytokines, and studies have shown that venlafaxine significantly reduced the mRNA expression of TNF-α, IL-6, IL-1β, and COX-2 (28) (Figure 1).

### Intestinal flora dysbiosis

Intestinal flora are normal microorganisms that reside in the human gastrointestinal tract for a long time and exist on the surface of the intestinal mucosal layer and in the intestinal lumen. These symbiotic flora synthesize a variety of vitamins essential for human growth and development, and also use protein residues to synthesize essential amino acids and participate in the metabolism of sugars and proteins, as well as promote the absorption of minerals such as iron, magnesium, and zinc and metabolize toxins (29). The brain and the gut are closely linked and various aspects of brain development, function, mood and cognition may be influenced by the symbiotic flora (30). The gut microbiota and CNS are connected through multiple bidirectional pathways involving neural, endocrine, and immune signaling, and dysbiosis of the gut flora can cause psychiatric disorders, such as depression (31–33). Current studies suggest that intestinal flora dysbiosis can mediate depression by causing an inflammatory response (34, 35). Inflammatory bowel syndrome (IBS) is a chronic, debilitating functional gastrointestinal disorder in which patients have impaired intestinal blood barrier integrity and leaky gut occurs, leading to the entry of intestinal contents such as immune cells and microbiota into the bloodstream, resulting in low-grade systemic inflammation, and circulating inflammation-related cytokines disrupt the blood-brain barrier, permitting infiltration of peripheral immune cells into the brain, leading to central inflammation in the brain, which central inflammation is associated with depression (36). Liu et al. (37) gave Chronic Unpredicted Mild Stress (CUMS) model rats Zhi Zhi Tang (ZZCD) for intervention and found that ZZCD promotes butyrate production by modulating the gut microbiota, and butyrate acts on the gut-brain axis, thereby It was found that ZZCD promotes butyrate production by regulating intestinal microbiota, and butyrate acts on the gut-brain axis, thus further regulating inflammation, neurotransmitters, endocrine and brain-derived neurotrophic factor (BDNF) to achieve antidepressant effects.

Intestinal barrier dysfunction is accompanied by increased intestinal permeability, permitting translocation of bacteria or their cell wall component LPS to the bloodstream, leading to persistent systemic inflammation (38). LPS is commonly used for modeling animal models of depression (17, 39). Rubab et al. (40) developed curcumin- nanostructured lipid carriers CUR-NLCs carriers (CUR-NLCs) and replicated the depression and anxiety model with LPS to study their neuroprotective effects in this model, and from histological and immunohistochemical analyses, CUR-NLCs improved brain tissue structure, inhibited the expression of p-NF-κB, TNF-α, and COX-2 in brain tissue, and enhanced the neuroprotective effects of curcumin, which could be used as a treatment for depression and anxiety as a potential drug. Natural drug substances can improve the levels of oxidative stress and neuroinflammation in depression, Muhammad et al. (41) used carvacrol to treat rats with depressive-like behavior induced by LPS, and significant neuronal alterations as well as elevated levels of inflammatory cytokines, such as TNF-α, COX-2 and c-Jun N-terminal kinase (p JNK). lPS stimulates microglia activation and inflammatory responses and upregulates mRNA and protein expression of inflammatory mediators such as COX-2 (42, 43). These inflammatory mediators can further mediate changes in the intestinal flora and disturbances in their metabolites, affecting brain function and depression. Estrogen deficiency can cause depression-like behavior, and Huang et al. (44) found in their experiments that estrogen deficiency-induced depression was associated with intestinal flora imbalance and inflammatory response in mice undergoing ovariectomy (OVX), where intestinal flora imbalance caused leaky gut and central inflammation (downregulation of COX-2 expression) to suppress BDNF expression in hippocampal neurogenesis, resulting in depressive behavior in OVX mice. BDNF is a neuroprotective molecule that plays a key role in neuroplasticity, neuroinflammation, learning and memory (45). There is growing evidence that modulation of BDNF expression levels can exert antidepressant effects (46–48), where BDNF-cAMP response element binding protein (CREB) signaling is one of the most attractive signaling pathways for the treatment of depression (49, 50). In summary, gut flora and COX-2-mediated inflammatory responses are strongly correlated, but there are fewer studies on how COX-2-mediated pathways and gut flora-brain centers interact and thus mediate depression, and further research is needed (Figure 1).

### Neurotransmitter disorders

To date, neurotransmitter dysregulation has an important role in the pathogenesis of depression. Among these, the traditional monoaminergic theory (serotonergic, noradrenergic and dopaminergic dysregulation) has received the most attention in the treatment of depression, and most of the available antidepressants target these monoaminergic systems, such as serotonin and/or noradrenergic reuptake inhibitors and antipsychotic compounds that antagonize dopamine D2 (51). However, the exact pathophysiological mechanisms underlying serotonergic, noradrenergic, and dopaminergic dysfunction in depression remain unclear, and it has been hypothesized that inflammatory mechanisms may be involved in the pathogenesis of depression.

5-hydroxytryptamine (5-HT) is a monoamine neurotransmitter, which is mainly distributed in the pineal gland and hypothalamus, and abnormalities in 5-HT levels and functions in the central nervous system may be associated with the development of various disorders such as psychosis and migraine. Studies have shown that 5-HT and the onset of depression are closely related (52, 53). Overproduction of inflammatory cytokines affects the function of the 5-HT system by inhibiting cellular inflammatory factors thereby decreasing IDO activity. IDO degrades tryptophan (TRP) in the blood, a precursor of 5-HT, and its decrease reduces 5-HT synthesis, therefore, increasing 5-HT synthesis by inhibiting the IDO/5-HT/TRP signaling pathway can promote and improve hippocampal cognitive dysfunction and exert anti-inflammatory and antidepressant effects (54, 55). COX-2, as an inflammatory cytokine, inhibition of COX-2 expression reduces oxidative stress, inflammatory factor release, upregulates 5-HTlA receptors in hippocampal tissue, ameliorates cortical inflammatory response damage, reestablishes monoamine transmitter system homeostasis, and attenuates depression-like behavior (56). COX-2 overexpression leads to an increase in the pro-inflammatory cytokines IL-1β, IL-6 and TNF, which increases the activity of the 5-HT transporter protein SERT, increases the affinity of the 5-HT receptor 5-HT1A, and mediates depression through an IDO mechanism that alters tryptophan metabolism (57). Su et al. (58) found that the acidic sphingomyelinase inhibitor acidic sphingomyelinase inhibitors blocked interferon-α-induced 5-HT uptake in T cells through a COX-2/Akt/ERK/stat-dependent pathway. Clinical trials and experimental studies have demonstrated that COX-2 inhibitors can produce adjuvant antidepressant therapeutic effects against selective 5-HT reuptake inhibitors (59), while chronic selective 5-HT reuptake inhibition improves the endothelium-dependent hyperpolarization-like pathway of the small arteries in the fraction of endothelial function and COX-2 levels, modulating endothelial dysfunction and oxidative status in a chronic mild stress model in depressed rats (60).

In addition to the monoamine transmitter 5-HT, the functions of other brain neurotransmitters such as histamine (HA), dopamine (DA), acetylcholine (ACh), gamma-aminobutyric acid (GABA), and glutamate are thought to be closely related to various brain disorders such as depression, schizophrenia, and anxiety disorders. Venkatachalam et al. (61) found that histamine H3R and dopamine D2R/D3R antagonist, ST-713, improved autistic-like behavior in BTBR T + tf/J mice by attenuating the protein expression levels of NF-κB p65, COX-2 and iNOS in the hippocampus and cerebellum of BTBR mice. Klawonn et al. (62) found that the COX-2/activation of the PGs/EPR pathway increased neuronal peak potential spacing, decreased membrane electrical resistance and posterior hyperpolarization (fAHP), and simultaneously inhibited the GABA and DA signaling pathways, which in turn inhibited DA transmitter production and transmission, thereby triggering negative mood and depression (61). Emerging preclinical and clinical findings suggest that maladaptive glutamatergic neurotransmission may underlie the pathophysiology of MDD and that the neurobiological mechanisms of stress-induced impairment of AMPA (α-amino-3-hydroxy-5-methyl-4-isoxazolepropionic acid)-glutamatergic neurotransmission in the brain may provide new insights into the pathogenesis of MDD (51). In summary, inhibition of the pro-inflammatory cytokine COX-2 in the inflammatory response has an effect on CNS and 5-HT, HA, GABA, DA and glutamatergic neurotransmission, further affecting the onset of depression, and this effect needs to be further evaluated, which should include clinical trials in a larger number of patients (Figure 1).

### Hyperfunction of the hypothalamic-pituitary-adrenal axis

The hypothalamic-pituitary-adrenal axis (HPA) axis is an important component of neuroendocrinology, and patients with depression often show hyperfunction of the HPA axis (63). Stress leads to hyperactivation of the HPA axis and disruption of the end product of HPA axis secretion, GC, which acts on glucocorticoid receptors (GR) in the hippocampus and hypothalamus, among other sites, after crossing the blood-brain barrier, causing disruption of the negative feedback regulation of the HPA axis, which in turn mediates the onset of depression (64–67). It has been shown that glucocorticoids and inflammatory responses are closely related and synthetic glucocorticoids (GCs) are widely used in a variety of inflammatory diseases, and the anti-inflammatory effects of GCs are achieved in part through the inhibition of a large number of pro-inflammatory cytokines (68, 69). Among them, GC correlate with the expression levels of the pro-inflammatory cytokine COX-2 (70, 71). The HPA axis acts as a negative feedback loop in which: stressors cause CRH production in the hypothalamus; CRH causes ACTH secretion in the pituitary; ACTH stimulates cortisol secretion in the adrenal cortex; and cortisol reversibly inhibits corticosteroid secretion (72). It has been shown that the HPA axis maintains the balance between cortisol and inflammation by regulating GR, and stress activates pro-inflammatory cytokines such as COX-2, which further stimulates the HPA axis to secrete cortisol (73), leading to depression (74, 75). overexpression of COX-2 leads to increased synthesis of prostaglandins, which in turn increases tissue sensitivity to catecholamines. stimulates the activity of the HPA axis through pro-adrenocorticotropin-releasing factors and leads to a surge in the production of pro-inflammatory cytokines (57). Acupuncture can exert anti-inflammatory effects through the HPA axis in order to reduce COX-2) and PGE2) levels and enhance the sympathetic nervous system (76). Chronic administration of corticosterone (CORT) in rodents is used to mimic stress-related dysregulation of the HPA axis and is a well-recognized approach in depression modeling, and Kv et al. (77) administered corticosterone to mice to replicate a depression model in which the pathogenesis of depression is increased levels of pro-inflammatory cytokines such as COX-2, which promotes GC resistance due to an overactive HPA axis, thereby increasing susceptibility to inflammatory responses that further leading to the development of depression. Therefore, COX-2 overexpression can affect the level of glucocorticoid and its receptor-mediated HPA axis function, which leads to the development of depression (Figure 1).

### Mitochondrial dysfunction

As an energy-supplying organelle of the body, mitochondria have numerous physiological functions such as synthesizing ATP, providing energy to cells, participating in the tricarboxylic acid cycle, oxidizing phosphate, and storing calcium ions. Studies have reported that inflammation negatively affects mitochondria, leading to excitotoxicity, oxidative stress, and energy deficit, and that dysfunctional mitochondria may drive or propagate the inflammatory response, thus creating a vicious cycle (78). Numerous basic studies in recent years have found that the onset of depression is associated with mitochondrial dysfunction in brain regions (79, 80). Mitochondrial dysfunction includes reduced ATP synthesis, oxidative respiratory chain dysfunction, abnormal mitochondrial structure and excessive occurrence of apoptosis (81).

It has been found that reduced mitochondrial function as well as impaired oxidative metabolism and COX expression levels in hippocampal tissue are closely linked, and the mechanism of action may be related to reduced mitochondrial membrane potential and cell death due to reduced energy production (82, 83). Metabolomic and proteomic analyses further revealed that disruption of mitochondrial energy metabolism is one of the pathophysiological symptoms of depression, and that changes in subunit proteins of cytochrome c, ATP synthase or NADH dehydrogenase and a decrease in oxidative phosphorylation due to reduced electron transport chain activity in chronic mild stress (CMS) model rats stimulate biochemical dysregulation in the process of ATP production (84). Wang et al. (85), based on the theory that mitochondrial dysfunction mediates the generation of depression, hypothesized that healthy mitochondrial transplantation could improve depressive symptoms, and their study demonstrated for the first time that mitochondrial transplantation produces antidepressant-like effects in lipopolysaccharide-induced depression. Isolated mitochondria significantly reduced hippocampal astrocyte and microglia activation and neuroinflammation (i.e., 1L-1β, TNF-α, and COX-2), increased BDNF expression, and restored ATP production dysfunction and oxidative stress in LPS-induced depression. Furthermore, impaired synaptic mitochondrial function in the hippocampus and amygdala has a key role in the pathophysiological impairment of depression, and scholars have studied the potential dysregulation of synaptic mitochondrial proteins in the hippocampus and amygdala of CMS rats based on proteomics coupled with mass spectrometry, respectively, and these proteins are associated with impaired oxidative phosphorylation of synaptic mitochondria in the hippocampus and impaired glutamatergic transmission in the amygdala, which can lead to synaptic morphological and functional abnormalities and, therefore, synaptic mitochondria may be a key therapeutic target during depression (86, 87). Studies have shown that non-ylphenol (NP) exposure can induce altered synaptic morphology, reduce learning and memory capacity, and increase the incidence of depression in fetal rats. Xu Weihong et al. (88) and Angrand et al. (89) studied depression-like behavior in rats by NPonly or NP + high-fat diets and showed that exposure to NP only or both NP and high-fat diets can lead to mitochondrial damage and dysfunction in hippocampal neurons. affecting synaptic morphological plasticity and COX-2 expression in the hippocampus, further leading to the development of depression. Therefore, disruption of mitochondrial energy metabolism, impairment of synaptic plasticity and altered COX-2 levels are key causes of the development of depression.

The occurrence of depression is associated with stressful stressful events, and stress leads to a disturbance in the redox homeostasis of the body (brain tissue is the main target), producing a state of oxidative stress, which is manifested by increased levels of reactive oxygen species (ROS) clusters in the body. Mitochondria are the main organelle for ROS production (90, 91) and an antioxidant defense system exists in mitochondria to repair oxidative damage, which helps to maintain cellular integrity. Under normal conditions, several antioxidant enzymes such as superoxide dismutase (SOD), catalase (CAT), glutathione peroxidise (GPx), and glutathione reductase (GR) maintain the physiological levels of ROS. However, when stimulated by external stresses such as stress, the mitochondrial redox buffering system is disrupted and oxidative stress occurs (92, 93). High levels of ROS are one of the hallmarks of mitochondrial oxidative stress. NADPH oxidase is an electron transport membrane protein that is mainly responsible for ROS production (94) and contributes to ROS production and COX/PGE expression in neuropsychiatric disorders (95). Inhibition of microglia NADPH oxidase activation and nitric oxide synthase reduces ROS and RNS production and exerts neuroprotective effects by ameliorating oxidative stress thereby achieving a therapeutic effect on depression (93). And studies have found that neuroinflammation, common in the brains of depressed patients, has a strong impact on ROS production, and there is evidence that inflammatory factors increase the risk of depression (96). Among these, there is a positive correlation between the expression level of the inflammatory factor COX-2 and ROS concentration (97–99). An increasing number of studies have shown that inflammation leads to increased ROS production and that mitochondrial oxidative stress plays an important role in the pathophysiology of depression (78, 100). In summary, mitochondrial dysfunction is one of the pathogenic mechanisms of depression, and mitochondrial dysfunction can affect COX-2 levels, which in turn affects depression by mediating neuroinflammation in the brain. In addition, studying the interaction between COX-2 and mitochondria facilitates the study of the mechanisms of neuroinflammatory response to depression (Figure 1).

### Hippocampal neuronal damage

Hippocampal microglia (MG) are the first line of defense of the CNS and are intrinsic immune cells in the brain, accounting for 5–12% of CNS cells, which regulate neuronal cell viability and dendritic spine density (101), provide neurological protection, and address inflammation through phagocytosis and tissue repair (102). Under normal conditions, MG is in a “resting” state and performs its immunosurveillance role. In contrast, changes in the cellular environment (e.g., oxidative stress leading to MG activation) alter its cellular morphology and function, and affect neuronal cell viability, function, and synaptic plasticity through exocytosis of inflammatory and other cytokines (103). MG activation is an important marker of hippocampal neuronal injury in depression.

Hippocampal neuronal damage is an important etiology mediating cognitive dysfunction and depression-like behavior (104). It has been reported that inflammation can induce neuronal apoptosis, impairment of synaptic plasticity and reduced excitability, among other impairments. As a typical pro-inflammatory factor, IL-1β induces neuronal apoptosis through the STAT3 pathway (105), while TNF-α and NO cause impaired neuronal viability through activation of the NF-κB pathway (106). Conversely, inhibiting the production of inflammatory factors or increasing the secretion of anti-inflammatory factors helps to maintain neuronal cell activity (107). In addition, inflammatory factors impair neuronal synaptic plasticity and thus cause abnormal neuronal function (108), e.g., IL-6 reduces neuronal synaptic plasticity and accelerates depression (109). MG is an important source of COX-2, and Strekalova et al. (57) found an increase in the number of iba-1 positive cells in the hippocampal region and a decrease in the number of ki67 positive cells in the granular subregion of depressed mice. positive cells, suggesting that MG activation in depressed mice inhibits cell proliferation, which in turn leads to overexpression of COX-2 in hippocampal CA1 areas as well as in dentate gyrus neurons, and that these changes mediate susceptibility to stress-induced lack of pleasure (a core symptom of depression). Recent studies have shown that activation of MG promotes the synthesis of more PGs by COX-2, which can act as a direct messenger factor between MG cells and neuronal cells (62). These PGs are secreted extracellularly by MG and further bind to prostaglandin receptors (EPR) on neuronal cell membranes, which in turn leads to decreased neuronal excitability and induces depression (61, 62, 110). It is thus evident that the inflammatory mediator CO can cause a decrease in neuronal excitability by mediating the synthesis of PGs, thus suggesting that inflammation is extensively involved in neuronal injury. Therefore, MG activation mediates COX-2/PGs pathway to decrease hippocampal neuronal excitability may be an important mechanism to trigger depression (62) (Figure 1).

## Cyclo-oxygen-ase-2 inhibitors

Cyclo-oxygen-ase-2 (COX-2) can regulate the occurrence and development of depression. In recent years, COX-2 inhibitors have been widely used in the treatment of depressed patients, and the drugs commonly used in clinical practice as COX-2 inhibitors are non-steroidal anti-inflammatory drugs (NSAIDs), all of which have varying degrees of efficacy in depression (111–113). Some natural compounds such as xanthines, alkaloids, astragals, flavonoids, terpenoids and quinones also have the ability to inhibit COX-2 activity.

### Non-steroidal anti-inflammatory agents

Cyclo-oxygen-ase-2 (COX-2) is widely involved in the inflammatory response and plays a crucial role in the inflammatory pathway, which can be improved by inhibiting it with NSAIDS, which can reduce the production of key pro-inflammatory mediators PG by inhibiting COX-1 and COX-2 (114). Long-term use of NSAIDS predisposes to gastrointestinal and cardiovascular diseases, which may be caused by gastric, renal, and platelet cell dysfunction due to COX-1 inhibition (115). Through research experts have found that selective COX-2 inhibitors have less side effects than non-selective COX-2 inhibitors. Among them, celecoxib is a selective COX-2 inhibitor, which is clinically the first-line drug in NSAID, Song et al. (116) found that CUMS exposure leads to increased COX-2 levels and that excess COX-2 converts arachidonic acid to PEG2 thereby leading to increased PEG2 expression, and that PEG2 production is associated with increased expression of the pro-inflammatory cytokines IL-1β, TNF a and IFN-γ (8), and celecoxib downregulates the production of these pro-inflammatory cytokines in the DG region, in addition to effectively attenuating mitochondrial ROS production and attenuating oxidative DNA oxidative damage, thereby potentially ameliorating the depressive phenotype resulting from neuroinflammation and apoptosis in the DG region of the hippocampus. Celecoxib also modulates COX-2 to improve indoleamine 2,3-dioxygenase activity, an enzyme that drives the metabolism of tryptophan and kynurenine in the CNS and degrades 5-HT (112, 117). The non-steroidal anti-inflammatory agent NS-398 is neuroprotective against excitotoxicity and neuroinflammatory injury, and it was found that NS-398 blocked the neuroinflammation-induced decrease in small albumin PVB in the prefrontal cortex reducing depressive effects (118). 5-LO, like COX-2, is a key enzyme in AA metabolism, and elevated levels of 5-LO and COX-2 expression accelerate inflammatory Inhibiting the inflammatory pathway arachidonic acid-cyclooxygenase-2/lipoxygenase (AA-COX-2/5-LO) pathway can regulate neurotransmitter metabolism, and the selective COX-2 inhibitor meloxicam is an inhibitor of this pathway, and Huang (119) gave meloxicam to CUMS rats, which improved cortical and hippocampal elevation of the biogenic amine neurotransmitters 5-HIAA, DOPAC and HVA in CUMS rats, which exert neuronal protective effects and inhibit inflammatory responses through inhibition of the AA/COX-2/5-LO pathway to achieve therapeutic effects in depression.

Diaryl-substituted heterocyclic compounds (e.g., thiazole, imidazole, triazole, oxazole, oxadiazole, pyrrole, thiophene) have been extensively studied as selective COX-2 inhibitors, and researchers have synthesized a series of COX-2 inhibitors with higher selectivity by improving the compounds, and thiazole derivatives can exert anti-inflammatory effects well as dual COX-2 and 5-LOX inhibitors. Zhang (120) synthesized a novel 5-aminothiazole ring-containing nuclear COX-2 inhibitor with higher anti-inflammatory effect than meloxicam. Hamoud MMS and other researchers (121) synthesized 1,3,4-oxadiazolyl derivatives (8a-g) and 1,2,4-triazolyl derivatives (10a,b and 11a-g), and through further experiments the newly designed compounds were found to have potent COX-2 inhibitory effects, possessing powerful with potent anti-inflammatory and antioxidant activities, capable of inhibiting TNF-α, NO and ROS production, and have the potential to be promising therapeutic agents for the treatment of inflammation-related psychiatric disorders. Novel chalcone compounds have excellent COX-2 inhibitory activity, can effectively suppress EGFR expression levels, and can overcome gastrointestinal side effects (122, 123). Novel COX-2 inhibitors 5-diaryl pyrrole nitrooxyethyl sulfide and related compounds, which could achieve potent anti-inflammatory effects with an overall safety profile and reduced gastrointestinal toxicity (124).

### New targeted preparation

In addition to improving the structure of compounds, experts have also enhanced the effectiveness of drugs as well as attenuated their side effects by creating novel formulations. Schmied et al. (125) used novel cellulose-based particles as adsorbent carriers to prepare celecoxib solid self-nanoemulsifying drug delivery system (SNEDDS), SNEDDS can improve the oral bioavailability and stability of low-soluble lipophilic drugs, enhance the release of their active ingredients, and further develop controlled release dosage forms. Sipos et al. (126) combined the NSAIDs meloxicam with novel nanomedicines to develop mucosal adherent nanoemulsions containing meloxicam, which has advanced drug transportability and stability to further increase the absorption and bioavailability of meloxicam in humans to systemic administration and nasal-to-brain delivery. They performed cytotoxic effects of the formulation on an NIH/3T3 mouse embryonic fibroblast cell line and the results supported the safe nasal suitability of the novel agent. Long-term administration of NSAIDs has adverse effects such as gastrointestinal, cardiovascular function and to ameliorate this side effect, some researchers designed celecoxib into a celecoxib a microsphere-microcrystal-gel delivery system for intra-articular injection using an ultrasound approach (127). Thus, the use of novel agents can improve the bioavailability and stability of NSAIDs and also attenuate their toxic side effects, but there are fewer studies on the use of modified new dosage forms of NSAIDs for the treatment of depression and further in-depth studies are needed.

### Natural products and their derivatives

Bangpungtongsung-san (BTS) is a Korean medicine with antidepressant effects consisting of 18 herbs. Park et al. (128) evaluated the antidepressant and anti-neuroinflammatory effects of BTS using an *in vivo* model of lisinopine-induced depression and an *in vitro* model of LPS-stimulated BV2 microglia. The results showed that BTS may exert antidepressant effects by affecting mood-related hormones, neuronal activity and neuroinflammation in lisinopine-induced depression in mice. *In vitro* experiments showed that BTS may achieve depression-inducing effects by downregulating Nos-2 and Cox-2 to inhibit NO production, stimulating pro-inflammatory cytokine production and inducing neuroinflammatory responses in microglia. Kaixin San (KXS) is a well-known herbal formula for treating depressed mood and improving various learning and memory disorders, which was found to have antidepressant effects in a previous study by Dong et al. (129). Further studies found that KXS can reduce glucocorticoid secretion by regulating the levels of serum cytokines (such as COX, IL-2, and TNF-α) while inhibiting HPA axis hyperactivity and inhibiting apoptosis and thus treating depression.

Studies have shown that herbal monomeric components have therapeutic effects on depression, and these monomeric components are the direct material basis for the efficacy of herbal medicines. Flavoprotein (FX) is an edible brown seaweed rich in natural carotenoids, and FX can prevent LPS-induced depression-like behavior in mice by modulating the AMPK-NF-κB signaling pathway and downregulating pro-inflammatory cytokines (IL-1β, IL-6, and TNF-α) as well as overexpression of iNOS and COX-2 in the hippocampus, frontal cortex, and hypothalamus (130). Dihydromyricetin (DMY) is an important flavonoid extracted from Daphyllostachys, and Wei et al. (131) found that DMY could inhibit neuroinflammation and improve LPS-induced depression-like behavior in mice by decreasing the secretion of pro-inflammatory factors TNFα, IL-6, IL-1β, COX-2, and iNOS *in vivo*. Trans-cinnamaldehyde (TCA) is an excellent CNS COX-2 inhibitor and is the main component of the traditional Chinese medicine Cinnamomum cassia. Lin et al. (132) constructed a depression model in BALB/c mice and gave TCA intervention treatment. TCA significantly decreased COX-2, TRPV1 and CB1 protein levels in the hippocampus, increased 5-HT levels in the hippocampus and decreased the Glu/GABA ratio. Studies have demonstrated that TCA has some effect in governing depression-like behavior, and these findings suggest that TCA treatment has antidepressant effects and that it mediates depression levels by mediating COX-2 levels and regulating neurotransmitters. Tao et al. (133) showed that luteolin containing PA methanolic extract mediates depression levels by balancing excitatory (glutamate) and inhibitory (GABA) brain monoamines, the voltage-gated ion channels (NaK/Ca-ATPase) and inhibition of NF-κB/TLR-4 pathway ameliorated neuroinflammation (TNF-α, IL-1β and COX-2) and improved seizure-complicated depression-like behavior in experimental epileptic mice. Catalpol, a highly active cyclic enol ether terpene glycoside, is a major constituent of Dictyostelium, which is rich, nutritious, and has excellent medicinal and nutritional value, and is reflected in classical clinical antidepressant formulas in Chinese medicine (134, 135). The antidepressant mechanism of catalpol involves ERK1/2/Nrf2 upregulation due to HO-1 activation and downregulation of factors related to the COX-2/iNOS/NO pathway suppressing neuroinflammation and triggering upregulation of the BDNF/TrkB pathway to enhance neurotrophy (136).

## Conclusion and future directions

The activation of the inflammatory response system is an important mechanism in the pathogenesis of depression, and patients with depression are associated with increased levels of central or peripheral inflammatory factors. COX is inextricably linked to the above hypothesis and pathological manifestations. However, the current research on COX-2 is mainly focused on tumor and cancer-related aspects, and the pathophysiological studies in central nervous system diseases such as depression are relatively weak. The evidence emphasizes that the inflammatory response mediated by COX-2 activation is a key pathological component of depression, and the use of COX-2 as a therapeutic target will become a new strategy for the prevention and treatment of depression in the future. Significantly improved depression in rats and mice, mainly by reducing the pro-inflammatory mediator PG and other pro-inflammatory factors associated with inflammation, thereby ameliorating neuroinflammation, intestinal flora disorders, neurotransmitter imbalance, mitochondrial dysfunction, and neuronal damage. In the pathogenesis of depression, these pathological links can in turn interact with each other to exacerbate the pathogenic process. Although animal and clinical experiments have shown positive effects of COX-2 inhibitors on these links, further experimental studies are needed to validate them in large clinical samples and to determine whether COX-2 inhibitors also exert antidepressant effects through other pathways. At present, the development of antidepressant applications of COX-2 inhibitors is still relatively weak. Clinically, some common COX-2 inhibitors can have side effects such as cardiovascular and gastrointestinal diseases after long-term use, thus, it is important to fully understand the role of COX-2 and its related pathways in the development of depression, and to use COX-2 inhibitors as lead compounds to synthesize compounds with high bioavailability, good stability performance, safety, and significant biological activity. This will provide a reliable basis for the development of antidepressant drugs by using COX-2 inhibitors as lead compounds to synthesize excellent compounds and dosage forms with high bioavailability, good stability, safety, and significant biological activity.

In recent years, scholars have discovered another novel isozyme of COX, which is a new structural isoform of COX derived from COX-1. Unlike COX-1, which is mainly found in vascular, gastric and renal tissues, COX-3 is expressed in the heart and brain, more in the brain, and it is widely involved in the anti-inflammatory pathway. the site of inhibition of inflammatory mediator PG by COX-3 is different, it mainly inhibits central nervous system sites and weakly inhibits peripheral PG synthesis, and one study found that COX-1 and COX-3 mRNA levels can induce depression by leading to impaired oxidative metabolism and reduced mitochondrial function (83). Thus, it is clear that COX-3 may be an important target for anti-inflammatory and antidepressant effects, but the related literature is poorly reported and needs to be explored in depth, and anti-inflammatory therapy against the target COX-3 would be another new avenue for depression treatment. In addition, different sites have different sensitivity to COX inhibitors. Hippocampus plays an important role in the development of depression, and hippocampal region is an important anatomical region associated with depression, while MG is the main source of COX, so the study of COX inhibitors in hippocampal MG may provide further new ideas and targets for the prevention and treatment of depression. Furthermore, the amygdala plays an important role in stress-related psychiatric disorders and may be affected in chronic stress (137, 138). Surprisingly, there is no information on the role of COX-2 in the amygdala in rodent studies, so further attention to COX-2 changes in the amygdala in rodent models could provide another new idea and target for the prevention and treatment of depression.

## Author contributions

YH drafted the manuscript and collected important background information. YSH and XL analyzed data and document. MZ and YW reviewed and revised manuscript. All authors contributed to the article and approved the submitted version.
